# Relationship of Epicardial Adipose Tissue Thickness with Early Indicators of Atherosclerosis and Cardiac Functional Changes in Obese Adolescents with Metabolic Syndrome

**DOI:** 10.4274/Jcrpe.1064

**Published:** 2013-09-18

**Authors:** Bedir Akyol, Mehmet Boyraz, Cevriye Aysoy

**Affiliations:** 1 Dr. Sadi Konuk Education and Research Hospital, Department of Pediatric Cardiology, İstanbul, Turkey; 2 Turgut Özal University Faculty of Medicine, Department of Pediatric Endocrinology and Diabetes, Ankara, Turkey; 3 Middle East Technical University, Department of Statistics, Ankara, Turkey

**Keywords:** metabolic syndrome, epicardial adipose, tissue, carotid intima-media thickness

## Abstract

**Objective:** Epicardial adipose tissue thickness (EATT) is suggested as a new cardiometabolic risk factor. Carotid intima-media thickness (IMT) is a potential indicator of subclinical atherosclerosis in patients with metabolic syndrome (MS). We investigated the association of EATT with carotid IMT and cardiac functional changes in obese adolescents with MS.

**Methods:** One hundred thirty-eight obese adolescents and 63 lean subjects were enrolled in the study. The obese subjects were divided into two subgroups based on the presence or absence of MS (MS group and non-MS group). All subjects underwent transthoracic echocardiographic examination for determination of left ventricular (LV) function, LV mass index (LVMI), and myocardial performance index (MPI). EATT and carotid IMT were also measured during echocardiography.

**Results:** The average LVMI measurements were higher in both MS and non-MS obese patients in comparison with the lean children. The MS group had significantly higher LVMI measurements than the non-MS and lean groups (88.5±23.0, 67.5±24.8 g/m2, and 62.4±18.2 g/m2, respectively; p<0.01). Carotid IMT was higher in both the MS and non-MS obese patients in comparison with the lean group. The MS group had significantly higher carotid IMT measurements than the non-MS and lean groups (0.91±0.23, 0.78±0.18, and 0.52±0.08 mm, respectively; p<0.01). The EATT was also increased significantly in patients with MS compared to lean adolescents (7.42±1.55 vs. 4.28±0.79mm; p=0.001). EATT was positively correlated with body mass index-SDS, waist circumference, fasting glucose, insulin, homeostasis model assessment-insulin resistance, triglyceride levels, LV thickness, LVMI, and MPI in the MS obese group. EATT was the only independent predictor of carotid IMT in the multivariate analysis (β= 0.69, p<0.001).

**Conclusion:** The findings of the present study demonstrate a close relationship of EATT with carotid IMT and early cardiac dysfunction in obese adolescents with MS. Assessment of EATT and carotid IMT in routine echocardiographic examinations is suggested as a feasible and reliable method for the evaluation of obesity with MS and its related cardiovascular risks in children and adolescents.

**Conflict of interest:**None declared.

## INTRODUCTION

Metabolic syndrome (MS) is defined as a combination of abdominal obesity, hypertension, dyslipidemia [high triglyceride (TG) and low and high-density lipoprotein (LDL and HDL) levels], and insulin resistance (IR) or impaired glucose tolerance. MS is not a disease of adults only but also has increasingly become a threat for children and adolescents. It is recognized, usually at ages fo llowing onset of puberty, by establishing presence of IR (1,2).

Patients with MS are at increased risk of cardiovascular disease (CVD). Subclinical atherosclerosis develops years before the clinical manifestations of CVD and therefore the identification of predictors of premature atherosclerosis is crucial. Carotid intima-media thickness (IMT) is an established surrogate marker of subclinical atherosclerosis in patients with MS (3,4,5). Ectopic fat is an important predictor of metabolic disease and CVD, carrying more risk than does general fat accumulation (6).

The epicardial adipose tissue thickness (EATT) represents cardiac and visceral adiposity, and it has been suggested as a new cardiometabolic risk factor (6). The epicardial adipose tissue is in direct contact with the myocardium, and it is highly active metabolically. The epicardial adipocytes can secrete a large number of cytokines and vasoactive peptides, including free fatty acids, interleukin-6, TNF-α, angiotensin II, and plasminogen activator inhibitor (7). All of these molecules can increase cardiovascular risk. Therefore, obese patients with MS should be monitored closely by measuring echocardiographic EATT. Although EATT has been related to IR, hypertension, and dyslipidemia, the association between EATT and certain surrogate markers of atherosclerosis such as carotid IMT is unclear (6,8,9).

MS has also been associated with left ventricular (LV) hypertrophy, LV diastolic dysfunction, and myocardial dysfunction (10). Voulgari et al (11) reported higher LV myocardial performance index (MPI) values in MS patients compared to normal subjects, indicating depressed ventricular functions. However, no data are available for obese adolescents with MS on the relationship of EATT with LV mass index (LVMI) and MPI.

In this study, we sought to examine the relationship of EATT with cardiac structural/functional changes and carotid IMT as observed by echocardiography. We also investigated the association of EATT with conventional metabolic and cardiovascular risk factors in obese adolescents with MS.

## METHODS

This study was conducted between September 2011 and November 2012 on patients presenting with the complaint of obesity to the Pediatric Endocrinology Department of the Sisli Etfal Education and Research Hospital in Istanbul. The study group included 138 (66 girls and 72 boys) obese pubertal adolescents aged between 9 and 18 years. Mean age was 13.4±3.69 years in the girls and 13.6±3.76 years in the boys. The control group consisted of 63 age-and sex-matched lean adolescents who were selected from cases referred for cardiac murmurs to the hospital but who were shown to have only innocent murmurs. The mean age of this group was 13.3±4.3 years (range: 9-18 years). Presence of anemia, type 1 or type 2 diabetes mellitus or other endocrinological disease, familial dyslipidemia or other chronic condition, psychiatric illness, or receiving medications known to effect insulin action or insulin secretion were reasons for exclusion from the study.

The diagnosis of MS was made according to the International Diabetes Federation (IDF) criteria. The IDF definition of MS for children aged 10 years or older includes a body mass index (BMI) > 90th percentile for age and sex and presence of two or more of the following findings: (1) TG >150 mg/dL; (2) HDL-cholesterol (HDL-Cho) <40 mg/dL; (3) systolic blood pressure >130 mmHg, diastolic blood pressure >85 mmHg; and (4) plasma glucose >5.6 mmol/L or >100 mg/dL or known type 2 diabetes (2).

The study was conducted in accordance with the guidelines proposed in the Helsinki Declaration and was approved by the Sisli Etfal Education and Research Hospital Ethics Committee on 19 July 2011. An informed consent form was obtained from the patients or the legal guardians.

Height and weight were measured in all subjects with an empty bladder in postabsorptive conditions. Height was measured to the nearest 0.5 cm using a standard height board, and weight was determined to the nearest 0.1 kg on a standard physician’s beam scale with the subject dressed only in light underwear without shoes. BMI was calculated by the weight (kg)/height (m^2^) formula. To compare BMI across different ages and in both boys and girls, BMI values were expressed as standard deviation scores (BMI-SDS). BMI-SDS was calculated using the Lambda, Mu, Sigma method, as described by Cole et al (12). Waist circumference (WC) was measured at the end of expiration between the midpoint of the last rib and superior iliac crest, using a non-stretch tape measure (13).

Pubertal development stage was assessed by the same pediatric endocrinologist according to Tanner criteria. Sexual maturation level was >2 in all patients (Tanner stages II–IV).

Blood pressure was measured by a mercury sphygmomanometer of appropriate size for age, following a minimum rest period of 10 minutes. The normal values for children established by the National High Blood Pressure Education Program Working Group were used as a reference to evaluate blood pressure measurements (14). A blood pressure value of ≥95th percentile for age, sex, and height was accepted as hypertension.

**Laboratory Analyses**

Blood samples were obtained in the morning after an overnight fast in all patients for determination of glucose, insulin, lipid profile [TG, total cholesterol (T-Cho), HDL-Cho, LDL-Cho, very-LDL-Cho (VLDL-Cho)], and high-sensitivity C-reactive protein (hs-CRP). The glucose oxidase method was used in the determination of blood glucose levels. Insulin levels were measured using a radioimmunoassay kit (Immunotech kit). Serum lipid profile was measured using a modular analytical system (Roche/Hitachi). Serum hs-CRP was determined using particle-enhanced immunoturbidimetry with latex microparticles sensitized with duck anti-CRP Immunoglobulin Y.

IR was analyzed using the homeostasis model assessment (HOMA-IR) based on the following formula: [fasting insulin (mIU/L) x fasting glucose (mmol/L)] /22.5. A HOMA-IR value greater than 3.16 was used to determine IR in pubertal patients (15,16).

**Echocardiographic Evaluation**

Transthoracic echocardiographic examinations were performed by one experienced pediatric cardiologist in all patients. Echocardiographic measurements were performed with a ViVid 7 Pro (GE Vingmed Ultrasound, Horten Norway). An electrocardiogram was taken from all subjects. The patients were studied without sedation while they were lying in the left lateral position. 3-MHz transducers were used in all echocardiographic studies. All possible echocardiographic windows obtained from the different Doppler devices such as two-dimensional, colored, pulsed-wave, continuous-wave, and pulsed-wave tissue were analyzed for the subjects lying supine or in the left lateral semi-recumbent position. LV systolic functions and LVMI were assessed using M-mode and 2D, whereas myocardial tissue rates and MPI were studied using tissue Doppler methods.

Conventional echocardiography measurements were performed according to the recommendations of the American Society of Echocardiography (17). The LV mass and the LVMI were calculated by using the method of Woythaler and his colleagues, which is also a modification of the method of Devereux and Reichek (18). According to this calculation, LV mass is 1.04 [(LV diameter at end diastole + end-diastolic interventricular septum thickness - LV posterior wall thickness at end-diastole)3 - (LV diameter at end diastole)3 - 13.6)], and LVMI is the LV mass/body surface area.

**Pulsed-Wave Tissue Doppler Imaging**

Pulsed Doppler and tissue Doppler were performed using a 3-MHz transducer. We measured early (E) and atrial (A) transmitral maximal flow velocities by pulsed-wave Doppler. Then, we calculated the ratio E/A. Early (E’) and late (A’) diastolic peak velocities were measured. The ratio of early and late diastolic annular velocities was calculated. Cardiac time intervals - isovolumic contraction time, isovolumic relaxation time, and systolic ejection time - were measured. Tissue Doppler derived by MPI was calculated as (isovolumic contraction time + isovolemic relaxation time)/systolic ejection time. E’ wave acceleration and deceleration times were also measured.

**Epicardial Adipose Tissue Thickness Measurements**

The thickness of the epicardial adipose tissue was measured from the right ventricular free wall in the parasternal long axis view. The epicardial adipose tissue was identified as an echo-free space in the pericardial layers on the two-dimensional echocardiography, and its thickness was measured perpendicularly on the free wall of the right ventricle at end diastole (19,20).

**Carotid Intima-Media Thickness Measurements**

Carotid IMT was measured, using a 7-MHz probe, from the common carotid artery at a point 5 mm proximal to its bifurcation, as often preferred in the literature (21). The transducer was placed perpendicular to the common carotid artery, and its long axis was adjusted parallel to the flow direction. Images obtained from the anterior wall were magnified threefold, and measurements were made with an electronic caliper. The measurements from three consecutive beats were averaged and recorded as the carotid IMT (22).

**Statistical Analysis**

A commercially available statistical package (SPSS for Windows Version 19.0; SPSS, Inc., Chicago, IL, USA) was used in the statistical analysis. Mean values were calculated for continuous variables. Absolute and relative frequencies were calculated for discrete variables. Univariate comparisons of continuous data were carried out with the use of unpaired student t-tests, and discrete variables were compared with chi-square test or Fisher’s exact test. The relationship between EATT and the other variables was assessed by using Pearson correlation analysis. Variables with a p-value of ≤0.05 on correlation analysis were entered in multivariate stepwise regression analysis with backward elimination. All comparisons were two sided and p<0.05 was considered to be significant.

## RESULTS

The characteristics of the study population are shown in Table 1. The obese adolescents were divided into two groups based on the presence or absence of MS, and 44 (31.8%) were found to show the criteria for MS diagnosis. The lean, non-MS and MS groups did not differ significantly in terms of age (13.3±4.3, 13.9±3.7, 13.3±4.1 year, respectively; p>0.05). However, the groups differed significantly in terms of BMI (20.1±1.3, 34.9±9.3, 38.4±4.0 kg/m2, respectively; p=0.001). The groups did not differ significantly with regard to heart rate (79±5 vs. 78±7 beats/min; p>0.05) or average hemoglobin value (13.1±1.04 vs. 12.9±1.1 g/dL; p>0.05).

The obese group with MS had significantly higher systolic and diastolic blood pressure values than the non-MS and the lean groups (p=0.0018). The MS obese group also had significantly higher T-Cho, TG, LDL-Cho, fasting glucose, insulin levels, and HOMA-IR than the non-MS obese and the lean groups, whereas HDL-Cho levels were lower in the MS obese group when compared to the non-MS and lean groups. BMI, BMI-SDS, WC, systolic and diastolic blood pressure values were significantly higher in the non-MS obese group when compared to the lean subjects. T-Cho, TG, LDL-Cho, fasting glucose and insulin levels were also significantly higher in the non-MS obese group when compared to leans, whereas HDL-Cho levels were lower in the non-MS obese group when compared to lean subjects. The lean group had lower HOMA-IR values than the both non-MS and MS obese groups.

Moreover, the MS obese group had significantly higher carotid IMT (0.91±0.23 vs. 0.78±0.18 vs. 0.52±0.08 mm), LVMI (88.5±23.0 vs. 67.5±34.8 vs. 62.4±18.2 g/m2), MPI (0.5±0.1 vs. 0.41±0.2 vs. 0.4±0.1), and EATT (7.38±1.76 vs. 6.42±1.55 vs. 4.28±0.79 mm) values as compared to the both non-MS and lean groups (Tables 1,2).

The mitral valve pulsed-wave Doppler analyses showed a statistically significant difference between the lean and MS groups in terms of diastolic early wave peak velocity (E’), diastolic late wave peak velocity (A’), (E’/A’), isovolumic relaxation time, isovolumic contraction values, average MPI, acceleration time and average deceleration time. The average isovolumic contraction values, isovolumic relaxation time, average MPI, and average deceleration time were greater in MS obese group than in non-MS obese group (p<0.05). The results of the tissue Doppler imaging studies from the mitral annulus for the three groups are also summarized in Table 2.

EATT (p=0.002, r=0.31) significantly correlated with carotid IMT in the MS group. Table 3 shows the correlation of carotid IMT with other clinical variables. The multivariate backward stepwise regression analysis demonstrated that EATT is the only independent predictor of carotid IMT in obese patients with MS (standardized β coefficient=0.65, p<0.001). Standardized β coefficient and p-value were 0.04 and 0.71 for BMI-SDS, 0.03 and 0.83 for WC, 0.19 and 0.39 for fasting glucose level, 0.10 and 0.29 for insulin level, 0.15 and 0.19 for HOMA-IR, 0.01 and 0.89 for systolic blood pressure, 0.07 and 0.51 for diastolic blood pressure, 0.05 and 0.77 for LV posterior wall diastolic thickness, 0.08 and 0.53 for LV posterior wall systolic thickness, 0.18 and 0.33 for LVMI, and 0.11 and 0.28 for MPI (Table 4).

## DISCUSSION

In the current study, we demonstrated the presence of asymptomatic cardiac dysfunction and an increased echocardiographic EATT and carotid IMT measurements in obese adolescents having MS. We also found that obese adolescents with MS had greater LVMI, carotid IMT, EATT, and MPI measurements as compared to both healthy lean adolescents and non-MS obese subjects. In obese adolescents with MS, EATT significantly correlated with metabolic and cardiac parameters such as BMI, WC, fasting insulin, IR, TG levels, hypertension, LV thickness, LVMI, MPI and carotid IMT.

Abdominal fat accumulation is associated with adverse cardiovascular and metabolic consequences not only in adults but also in children and adolescents, as many pro-inflammatory cytokines are predominantly secreted from visceral adipose tissue. Abdominal fat is considered to be a better predictor of cardiovascular risk than BMI. Visceral obesity is most commonly determined in children by the measurement of WC and by plotting it on age and gender specific charts (23). In our study, we found a positive correlation between epicardial fat and WC in obese adolescents. This suggests that also in adolescents, epicardial fat can be used as an easy, accurate and inexpensive method for evaluation of visceral fat.

We also observed a high correlation between BMI-SDS and EATT. Our findings were similar to those reported by Abaci et al (24). Increased thickness of epicardial tissue was also reported in growth hormone-deficient adolescents who had increased BMI and WC values (25). Mazur et al (26) reported a close relationship of EATT with BMI and WC in obese children. We did not find other publications on EATT in children in the available literature, and data on such a correlation in adults are not consistent.

In our MS subjects, there was a statistically significant relationship between epicardial fat and IR as measured by HOMA index. This is in accordance with the results of Abaci et al (24) who reported a correlation between EATT and parameters of lipid and glucose metabolism. These authors suggested that a 4.1-mm cut-off value for EATT might be used as a simple screening method predicting IR in obese children.

MS represents a cluster of risk factors associated with an increased risk of life-threatening metabolic and cardiovascular complications of obesity. Ahn et al (27) showed that average EATT was higher in patients with unstable ischemic heart disease. This finding can be due to the fact that epicardial fat can be a source of inflammation, which might increase the risk of cardiac ischemia. Unlike the results reported by Mazur et al (26), in our study, we found a significant difference in EATT between obese children with and without MS. We were not able to find other comparable studies in obese children with MS, but in adults. Okyay et al (28) reported a close relationship between EATT and MS. In that study, a cut-off point of 4.35 mm determined MS with 61.7% sensitivity and 79.2% specificity. Ahn et al (27) demonstrated that patients with MS had thicker EATT than those without it. The thickness increased with an increasing number of components of MS. In another study, adults with IR and low adiponectin levels had the highest EATT values, independently of BMI (29). These data showed that in adults, epicardial fat might be used as a marker of visceral obesity, of IR and MS.

In our study, we found that echocardiographic EATT closely correlated with carotid IMT in patients with MS. This association was more peculiar than the other risk factors including WC in these patients. Therefore, we hypothesize that echocardiographic EATT might be a better indicator of premature atherosclerosis than WC in patients with MS. As such, MS patients with increased EATT on routine echocardiograms should be further evaluated for the presence of CVD. Indeed, EATT reflects visceral fat tissue and obesity and has been suggested as a new cardiometabolic risk factor (7,8,9). Aydin et al (30) have demonstrated that EATT indicated endothelial dysfunction in patients with MS. Natale et al (31) found that EATT also reflected the carotid artery stiffness in patients with hypertension. Moreover, in recent studies, EATT is reported to be a marker for presence and severity of coronary artery disease (32,33). Our study supports these results and suggests the possible association of EATT with subclinical atherosclerosis. Although BMI, WC and hip circumference, fasting glucose and insulin levels, HOMA-IR, HDL-Cho, TG levels, LV thickness, LVMI, MPI and carotid IMT were the correlates of EATT in our study population, EATT was the only independent variable that was significantly associated with carotid IMT in multivariate logistic regression analysis.

Magnetic resonance imaging (MRI) and computed tomography (CT) are more precise methods for quantifying visceral adiposity and EATT; however, these modalities are relatively more expensive than echocardiography. In addition, the radiation risk of CT limits its use as a screening tool. On the other hand, compared to CT and MRI, echocardiography is simple, rapid, safe, and low cost. The visceral fat thickness measured by ultrasonography is associated with increased carotid IMT in diabetic patients with normal WC values (34). Liu et al (35) demonstrated that mesenteric fat thickness was an independent determinant of MS and identified subjects with increased carotid IMT. Nelson et al (36) found that EATT ≥5.0 mm could identify an individual having detectable carotid atherosclerosis with a higher likelihood. Similarly, we observed that EATT measured by transthoracic echocardiography reflects the carotid IMT, which in turn, can help identify MS patients with subclinical atherosclerosis. Atherosclerosis has a long asymptomatic phase, and early treatment can reduce the risk of coronary events or stroke. The findings from our study suggested that EATT could be a more reliable indicator of subclinical atherosclerosis than other parameters such as WC in patients with MS. The EATT, which is easily measured during routine echocardiographic examination, can help select patients with MS who may benefit from advanced diagnostic studies and aggressive lifestyle changes.

The cardiac structural changes in obesity such as increased LV mass or systolic and diastolic dysfunction have already been supported by previous data (37,38). EATT could be a marker of cardiac adiposity and obesity; therefore, it could play an important role in obesity-related LV abnormalities. EATT was also found to correlated with LVMI, systolic and diastolic function in obese adolescents with MS. The close anatomical and functional relationship of EATT with the adjacent myocardium could lead to local and paracrine interactions between these tissues (39). MPI is a new echocardiographic parameter that correlates well with invasive measurements and can be used to evaluate both systolic and diastolic functions (40). In our study, we showed for the first time that echocardiographic measurements of EATT significantly correlated with LVMI and systolic and diastolic function in obese adolescent with MS.

In conclusion, the present study has shown that the measurement EATT by transthoracic echocardiography had a strong correlation with the carotid IMT, a surrogate marker of subclinical atherosclerosis, and could be considered a better index than WC in obese patients with MS. The echocardiographic assessment of epicardial fat may have the potential to be a simple marker for diagnosis of subclinical atherosclerosis and assessment of increased cardiovascular risk in patients with MS. Future research is necessary on larger series with accurate measurements of epicardial fat volume. Finally, it is also important to measure the adipokines to find a causal link between EATT and carotid IMT in patients with MS.

## Figures and Tables

**Table 1:**
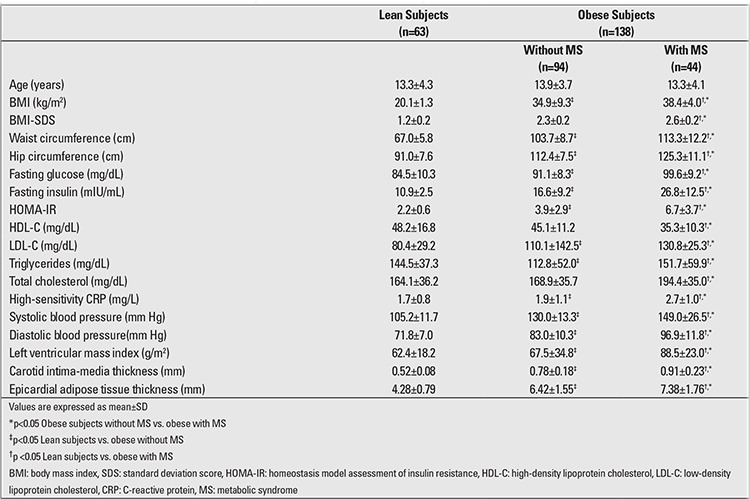
Characteristics of the lean and obese subjects with and without metabolic syndrome (MS)

**Tablo 2:**
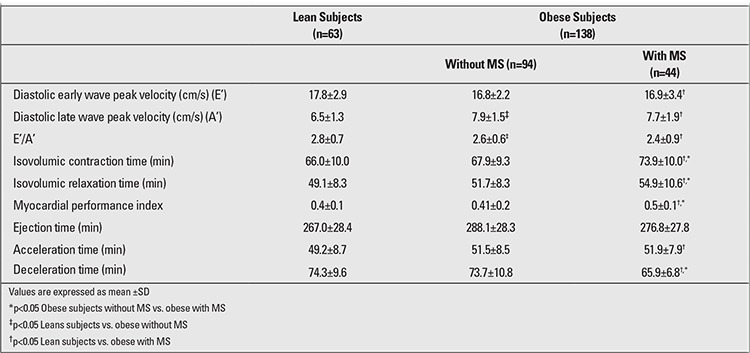
Tissue Doppler echocardiography measurements in the lean subjects and in the non- metabolic syndrome (MS) and MS obese subjects

**Table 3:**
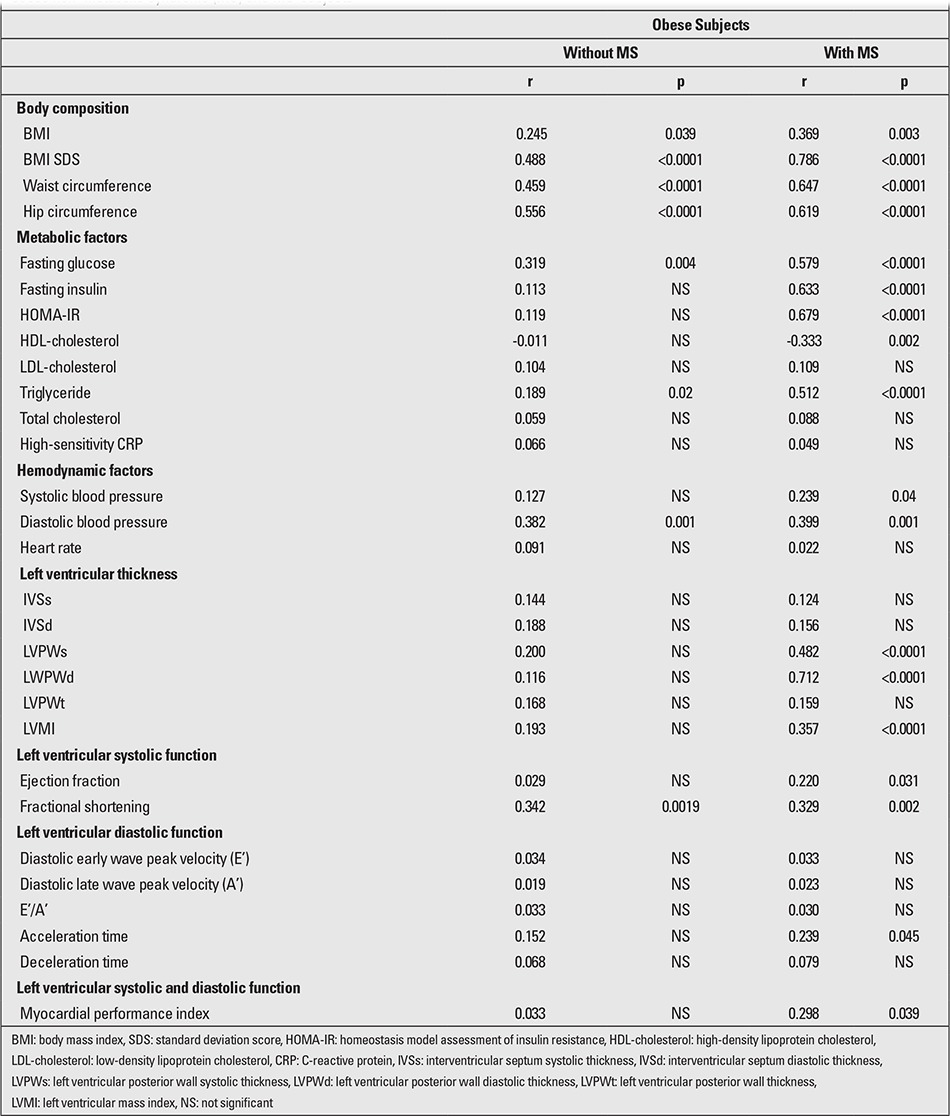
Pearson’s correlation coefficient between epicardial adipose tissue thickness (EATT) and metabolic/ echocardiographic parameters in the obese non- metabolic syndrome (MS) and MS subjects

**Tablo 4:**
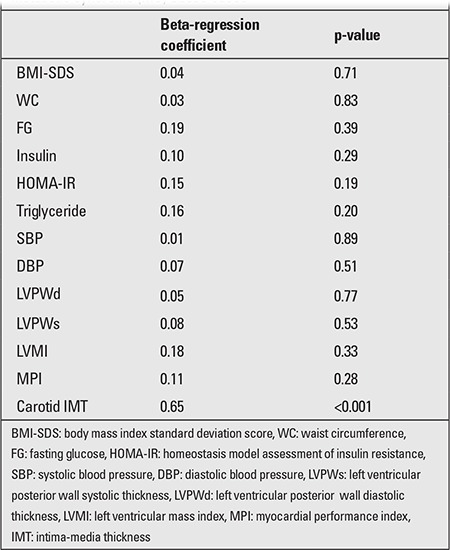
Independent predictors for epicardial adipose tissue thickness (EATT) by multivariate backward stepwise regression analysis in metabolic syndrome (MS) obese cases
